# Supplementary material for: CD3-engaging bispecific antibodies trigger a paracrine regulated wave of T-cell recruitment for effective tumor killing
Source: Commun Biol. 2024 Aug 13;7:983. doi: 10.1038/s42003-024-06682-9 (PMC11322607; doi:10.1038/s42003-024-06682-9)
Supplement: Supplementary file 2 — Supplementary Information [file 42003_2024_6682_MOESM2_ESM.pdf]

CD3-engaging bispecific antibodies trigger a paracrine regulated wave of T-cell recruitment for effective tumor killing

Chen-Yi Liao[1], Patrick Engelberts[2], Andreea Ioan-Facsinay[2], Janna Eleonora Klip[1], Thomas Schmidt[3], Rob Ruijtenbeek[2], Erik HJ Danen[1]

[1]Leiden Academic Centre for Drug Research, Leiden University, Leiden NL; [2]Genmab; Utrecht, the Netherlands; [3]Leiden Institute of Physics, Leiden University, Leiden NL.

Correspondence to: E.H.J. Danen (e.danen@lacdr.leidenuniv.nl)

Supplemental figures and figure legends

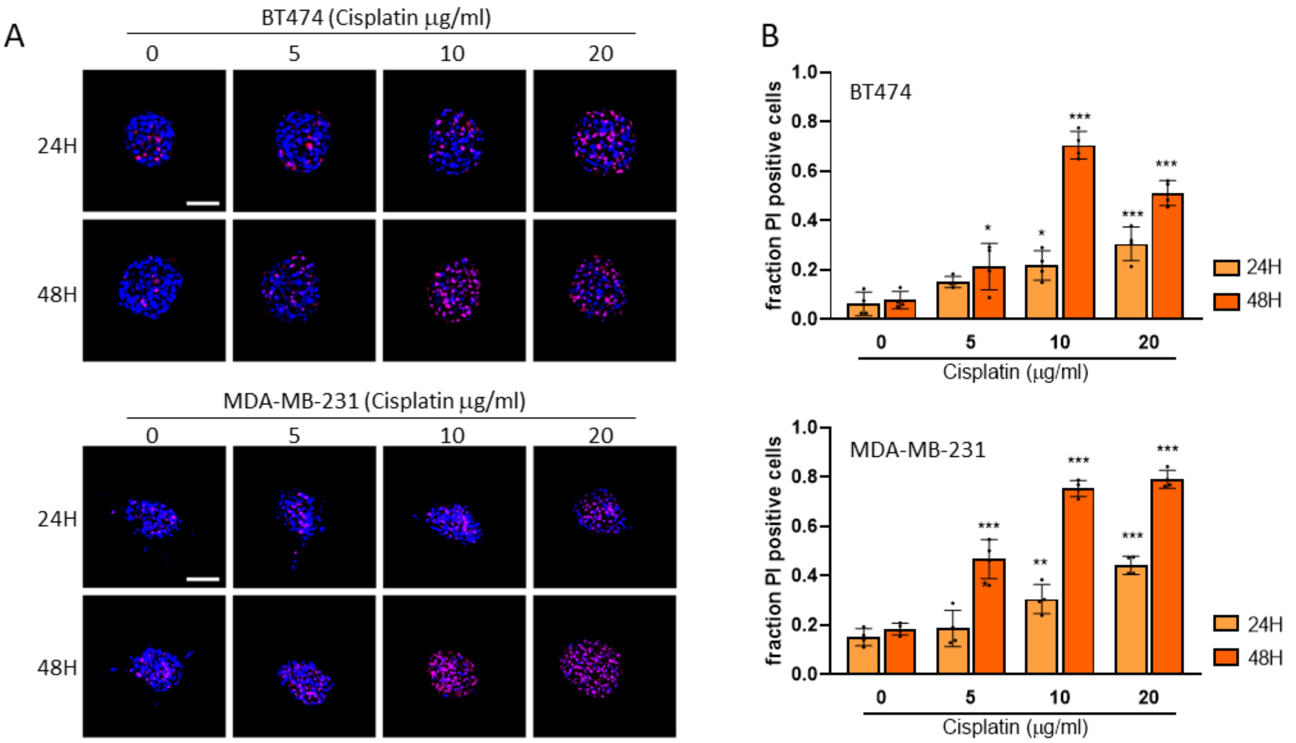

Supplementary Figure 1. Quantitative image-based analysis of viability of ECM embedded tumoroids. **(A)** Tumoroids were printed in collagen gels and exposed to a concentration range of cisplatin. Confocal image stacks were captured at 24 and 48 hours. Images show maximum projections. Bar = 100 μm. Blue, Hoechst; Red, PI. **(B)** Quantification of the image data as shown in A. Graphs show mean and SEM of 3 independent experiments, each performed in triplicate (3 individual wells each containing 1 collagen embedded BT474 or MDA-MB-231 tumoroid). Image quantification was performed on each individual z-section using the image analysis algorithm described in Fig 1B. P-value calculated using two-way ANOVA followed by Tukey's multiple comparisons test. *, $P < 0.05$; ***, $P < 0.001$, compared to untreated tumoroids.

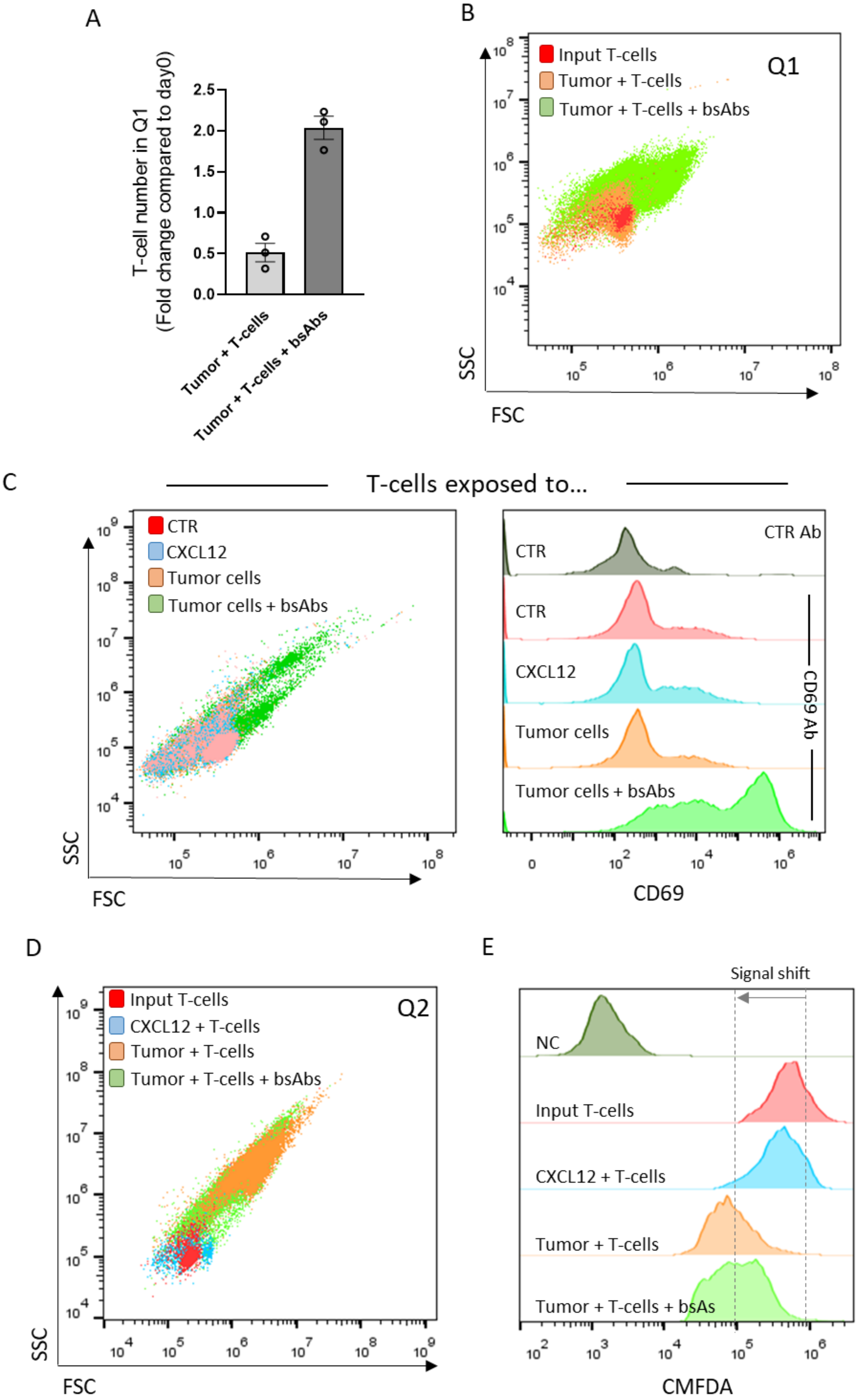

A
T-cell number in Q1
(Fold change compared to day0)
2.5
2.0
1.5
1.0
0.5
0.0
Tumor + T-cells
Tumor + T-cells + bsAbs
B
Input T-cells
Tumor + T-cells
Tumor + T-cells + bsAbs
Q1
SSC
FSC
C
T-cells exposed to...
CTR
CXCL12
Tumor cells
Tumor cells + bsAbs
SSC
FSC
CTR Ab
CTR
CXCL12
Tumor cells
Tumor cells + bsAbs
CD69 Ab
CD69
D
Input T-cells
CXCL12 + T-cells
Tumor + T-cells
Tumor + T-cells + bsAbs
Q2
SSC
FSC
E
Signal shift
NC
Input T-cells
CXCL12 + T-cells
Tumor + T-cells
Tumor + T-cells + bsAs
CMFDA

Supplementary Figure 2. Signs of proliferation and activation of T-cells in the presence of bsAbs. **(A)** The number of T-cells in Q1 (unlabeled T-cells co-cultured with tumor cells in the bottom chamber in absence or presence of $CD3_{wt}xHER2_{169}$ bsAbs; see Fig 7C), normalized to day 0. Mean and SEM from 3 biological replicates are shown. **(B)** Plot showing forward scatter (FSC) versus side scatter (SSC) of T-cells in Q1 of Fig 7C. Input T-cells were collected before being added to the lower compartment. **(C)** FSC/SSC plot (left) and CD69 staining (right) for T-cells exposed to the indicated stimuli. **(D)** Plot showing FSC versus SSC of CMFDA-labeled T-cells from Q2 (labeled T-cells recruited from the upper chamber; see Fig 7C). Input T-cells were collected after CMFDA labeling and before being added to the upper chamber. **(E)** CMFDA intensity of T-cells from Q2. NC, T-cells not labeled with CMFDA.

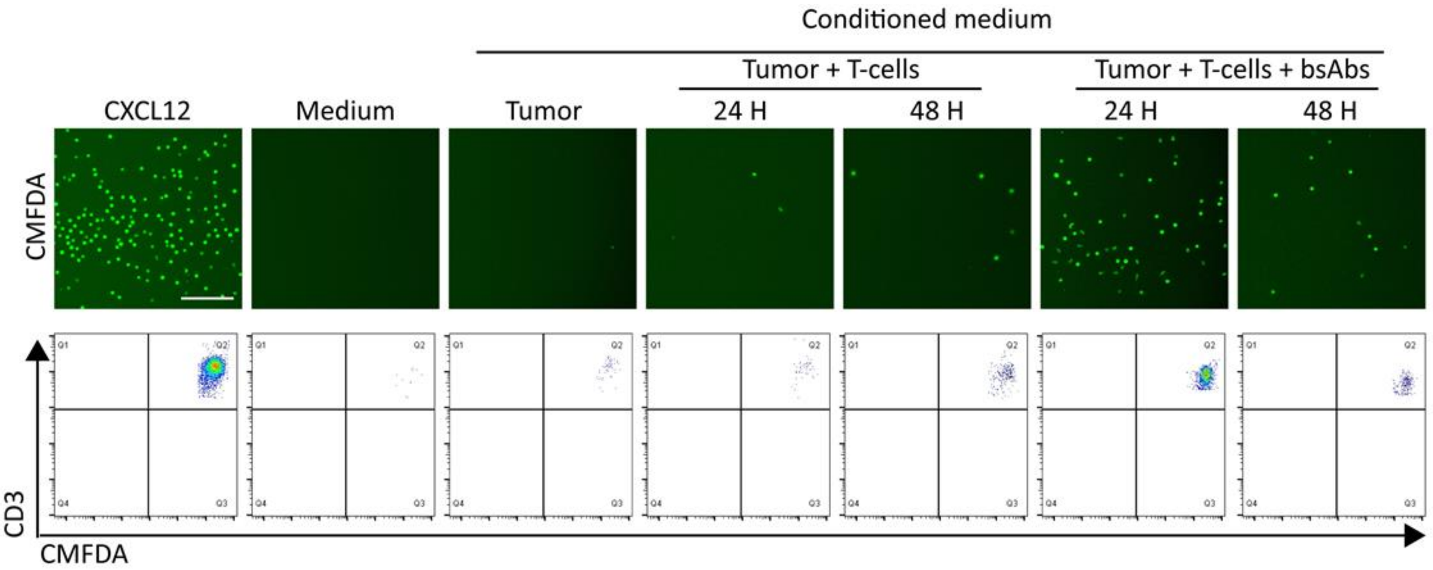

Supplementary Figure 3. Transwell assay for T-cell migration in the presence of CXCL12, control medium, or conditioned media obtained from BT474 cells alone or BT474 cells cultured in the presence of T-cells with or without $CD3_{wt}xHER2_{169}$ bsAbs. Top row shows green fluorescence channel images taken in the lower chamber after 48h (CMFDA-labeled T-cells migrated from the upper to lower compartment). Bar = 100 µm. Bottom row shows plots with CMFDA versus CD3 labeling of the migrated T-cells acquired from the lower chamber.
