# Supplementary material for: CD3-engaging bispecific antibodies trigger a paracrine regulated wave of T-cell recruitment for effective tumor killing
Source: Commun Biol. 2024 Aug 13;7:983. doi: 10.1038/s42003-024-06682-9 (PMC11322607; doi:10.1038/s42003-024-06682-9)
Supplement: Supplementary file 3 — Description of Additional Supplementary Files [file 42003_2024_6682_MOESM3_ESM.pdf]

Description of Additional Supplementary Files

Supplementary Movie 1. Time-lapse videos showing T-cell recruitment and tumor killing in BT474 tumoroids treated with 0.1 μg/mL $CD3_{wt}xHER2_{Herceptin}$ **(left)** and $CD3_{wt}xHER2_{LbD2}$ **(right)** bsAbs. Videos show maximum projections of confocal stacks and run from day 2 to day 5 with 1 hour interval. Blue, tumor nucleus; Green, T-cells; Red, PI.

Supplementary Movie 2. Time-lapse videos showing T-cell recruitment and tumor killing in BT474 tumoroids treated with 1 μg/mL $CD3_{wt}xHER2_{169}$ **(left)** and $CD3_{wt}xHER2_{153}$ **(right)** bsAbs. Videos show maximum projections of confocal stacks and run for 72 hours with 1 hour interval starting at the moment of initial T-cell-tumoroid contact. Blue, tumor nucleus; Green, T-cells; Red, PI.

Supplementary Movie 3. Time-lapse videos showing T-cell recruitment and tumor killing in BT474 tumoroids treated with 1 μg/mL $CD3_{wt}xHER2_{169}$ **(A)** and $CD3_{wt}xHER2_{153}$ **(B)** bsAbs. Videos show confocal images of a single z-section through the center of the tumoroid and run for 8 hours with a 1-minute interval starting at 36 hours after addition of T-cells and bsAbs. Red arrow indicates T-cells that are attracted to the site of T-cells initially interacting with the tumoroid. Blue, tumor nucleus; Green, T-cells; Red, PI.

Supplementary Data

The source data underlying the graphs in the figures.
