# Supplementary material for: CD3-engaging bispecific antibodies trigger a paracrine regulated wave of T-cell recruitment for effective tumor killing
Source: Commun Biol. 2024 Aug 13;7:983. doi: 10.1038/s42003-024-06682-9 (PMC11322607; doi:10.1038/s42003-024-06682-9)
Supplement: Supplementary file 9 — Reporting Summary [file 42003_2024_6682_MOESM9_ESM.pdf]

nature portfolio

Corresponding author(s): Erik Danen COMMSBIO-24-2462A

Last updated by author(s): Jul 17, 2024

Reporting Summary

Nature Portfolio wishes to improve the reproducibility of the work that we publish. This form provides structure for consistency and transparency in reporting. For further information on Nature Portfolio policies, see our Editorial Policies and the Editorial Policy Checklist.

Statistics

For all statistical analyses, confirm that the following items are present in the figure legend, table legend, main text, or Methods section.

n/a	Confirmed	
☐	☒	The exact sample size (*n*) for each experimental group/condition, given as a discrete number and unit of measurement
☐	☒	A statement on whether measurements were taken from distinct samples or whether the same sample was measured repeatedly
☐	☒	The statistical test(s) used AND whether they are one- or two-sided<br>*Only common tests should be described solely by name; describe more complex techniques in the Methods section.*
☐	☒	A description of all covariates tested
☐	☒	A description of any assumptions or corrections, such as tests of normality and adjustment for multiple comparisons
☐	☒	A full description of the statistical parameters including central tendency (e.g. means) or other basic estimates (e.g. regression coefficient) AND variation (e.g. standard deviation) or associated estimates of uncertainty (e.g. confidence intervals)
☐	☒	For null hypothesis testing, the test statistic (e.g. *F*, *t*, *r*) with confidence intervals, effect sizes, degrees of freedom and *P* value noted<br>*Give P values as exact values whenever suitable.*
☒	☐	For Bayesian analysis, information on the choice of priors and Markov chain Monte Carlo settings
☒	☐	For hierarchical and complex designs, identification of the appropriate level for tests and full reporting of outcomes
☒	☐	Estimates of effect sizes (e.g. Cohen's *d*, Pearson's *r*), indicating how they were calculated

Our web collection on statistics for biologists contains articles on many of the points above.

Software and code

Policy information about availability of computer code

Data collection	no code used for data collection
Data analysis	ImageJ 1.53c, CellProfiler version 2.2.0, MatLab R2018a, FlowJo (version 10), GraphPad Prism 8

For manuscripts utilizing custom algorithms or software that are central to the research but not yet described in published literature, software must be made available to editors and reviewers. We strongly encourage code deposition in a community repository (e.g. GitHub). See the Nature Portfolio guidelines for submitting code & software for further information.

Data

Policy information about availability of data

All manuscripts must include a data availability statement. This statement should provide the following information, where applicable:

- Accession codes, unique identifiers, or web links for publicly available datasets
- A description of any restrictions on data availability
- For clinical datasets or third party data, please ensure that the statement adheres to our policy

All data generated or analyzed during this study are included in this published article and its supplementary information files.

Human research participants

Policy information about studies involving human research participants and Sex and Gender in Research.

Reporting on sex and gender	N/A
Population characteristics	N/A
Recruitment	N/A
Ethics oversight	N/A

Note that full information on the approval of the study protocol must also be provided in the manuscript.

Field-specific reporting

Please select the one below that is the best fit for your research. If you are not sure, read the appropriate sections before making your selection.

☒ Life sciences ☐ Behavioural & social sciences ☐ Ecological, evolutionary & environmental sciences

For a reference copy of the document with all sections, see nature.com/documents/nr-reporting-summary-flat.pdf

Life sciences study design

All studies must disclose on these points even when the disclosure is negative.

Sample size	For all experiments cell sample sizes were chosen to create tumoroids of equal size
Data exclusions	no data were excluded
Replication	data were collected from at least 3 independent experiments, each performed in triplicate
Randomization	N/A
Blinding	N/A

Reporting for specific materials, systems and methods

We require information from authors about some types of materials, experimental systems and methods used in many studies. Here, indicate whether each material, system or method listed is relevant to your study. If you are not sure if a list item applies to your research, read the appropriate section before selecting a response.

Materials & experimental systems

n/a	Involved in the study	
☐	☒	Antibodies
☐	☒	Eukaryotic cell lines
☒	☐	Palaeontology and archaeology
☒	☐	Animals and other organisms
☒	☐	Clinical data
☒	☐	Dual use research of concern

Methods

n/a	Involved in the study	
☒	☐	ChIP-seq
☐	☒	Flow cytometry
☒	☐	MRI-based neuroimaging

Antibodies

Antibodies used	primary antibodies against Her2 (MA5-14057, e2-4001, 3B5, Thermo Scientific; 2165T, Cell Signaling), tubulin (T9026, DM1A, Sigma-Aldrich), Horseradish peroxidase (HRP)-conjugated secondary antibodies (Jackson Immunoresearch, anti-rabbit 111-035-003; anti-mouse 115-035-003). AlexaFluor-647 conjugated anti-rabbit secondary antibody (111-605-003, Jackson). Brillian Violet 421TM anti-human CD3 antibody (317343; BioLegend, San Diego CA, USA). PE-Cyanine7 anti-human CD69 antibody (310911; BioLegend, San Diego CA, USA). CD3xHer2 bsAbs (Genmab)
Validation	The primary and secondary antibodies were validated by manufacturers. The bsAbs were validated by Genmab and published previously.

Eukaryotic cell lines

Policy information about cell lines and Sex and Gender in Research

Cell line source(s)	American Type Culture Collection (ATCC)
Authentication	cell lines authenticated using STR profiling
Mycoplasma contamination	all cell lines tested negative for mycoplasma
Commonly misidentified lines (See ICLAC register)	N/A

Flow Cytometry

Plots

Confirm that:

☒ The axis labels state the marker and fluorochrome used (e.g. CD4-FITC).

☒ The axis scales are clearly visible. Include numbers along axes only for bottom left plot of group (a 'group' is an analysis of identical markers).

☒ All plots are contour plots with outliers or pseudocolor plots.

☒ A numerical value for number of cells or percentage (with statistics) is provided.

Methodology

Sample preparation	samples were collected from lower chambers of transwell assay and incubated with anti-human CD3 antibody to indentify T cell population.
Instrument	CytoFLEX, Beckman Coulter
Software	FlowJo version 10
Cell population abundance	The cell populations in the lower chamber contained CellTracker-stained T cells, unstained T cells, and tumor cells.
Gating strategy	T cells were first indentified and seperated from tumor cells based on CD3 antibody staining, T cells without CD3 staining provided as negative control. Subsequently, CellTracker CMFDA was used to indentify the T cells that migrated from the upper chamber.

☒ Tick this box to confirm that a figure exemplifying the gating strategy is provided in the Supplementary Information.
